# Telomere elongation protects heart and lung tissue cells from fatal damage in rats exposed to severe hypoxia

**DOI:** 10.1186/s40101-018-0165-y

**Published:** 2018-02-17

**Authors:** Yaping Wang, Zhen Zhao, Zhiyong Zhu, Pingying Li, Xiaolin Li, Xiaohong Xue, Jie Duo, Yingcai Ma

**Affiliations:** Department of Digestion, Qinghai Provincial People’s Hospital, Xining, Qinghai 810007 China

**Keywords:** Hypoxia, Heart and lung tissue cells, Telomere, Telomerase reverse transcriptase, Hypoxia-inducible factor, Reactive oxygen species

## Abstract

**Background:**

The effects of acute hypoxia at high altitude on the telomere length of the cells in the heart and lung tissues remain unclear. This study aimed to investigate the change in telomere length of rat heart and lung tissue cells in response to acute exposure to severe hypoxia and its role in hypoxia-induced damage to heart and lung tissues.

**Methods:**

Forty male Wistar rats (6-week old) were randomized into control group (*n* = 10) and hypoxia group (*n* = 30). Rats in control group were kept at an altitude of 1500 m, while rats in hypoxia group were exposed to simulated hypoxia with an altitude of 5000 m in a low-pressure oxygen chamber for 1, 3, and 7 days (*n* = 10). The left ventricular and right middle lobe tissues of each rat were collected for measurement of telomere length and reactive oxygen species (ROS) content, and the mRNA and protein levels of telomerase reverse transcriptase (TERT), hypoxia-inducible factor1α (HIF-1α), and hypoxia-inducible factor1α (HIF-2α).

**Results:**

Increased exposure to hypoxia damaged rat heart and lung tissue cells and increased ROS production and telomere length. The mRNA and protein levels of TERT and HIF-1α were significantly higher in rats exposed to hypoxia and increased with prolonged exposure; mRNA and protein levels of HIF-2α increased only in rats exposed to hypoxia for 7 days. TERT was positively correlated with telomere length and the levels of HIF-1α but not HIF-2α.

**Conclusions:**

Acute exposure to severe hypoxia causes damage to heart and lung tissues due to the production of ROS but promotes telomere length and adaptive response by upregulating TERT and HIF-1α, which protect heart and lung tissue cells from fatal damage.

## Background

Hypobaric hypoxia at high altitude can be particularly detrimental to human health. It has been well documented that acute exposure to hypoxia at high altitude will cause serious damage to a wide variety of human organs, in particular the heart and lung tissues. High-altitude pulmonary edema and heart disease are the most common high-altitude-related illnesses in otherwise healthy lowlanders at altitudes typically above 2500 m, with a high mortality rate in the absence of adequate treatment [1, 2]. However, the prevention and treatment of high-altitude-related heart and lung diseases remain challenging.

It is known that acute exposure to severe hypoxia at high altitude could induce adaptive response of cells to hypoxia, and damage such as cellular senescence and apoptosis can occur when cells no longer have to ability to withstand hypoxia. Telomere shortening is a well-established cause of cellular senescence and apoptosis that can be induced by many intrinsic and extrinsic factors, and telomere length is positively related to the life span of cells. Shortening and/or damage to telomeres can accelerate the damage response of cells and consequently result in cellular senescence and apoptosis [3–6]. However, considerable controversy remains regarding the effect of hypoxia on the telomere length. Some reports showed that hypoxia upregulated telomerase activity, resulting in elongation of telomeres and consequently extension of the life span of vascular smooth muscle cells [7], while some other studies showed that oxidative stress could induce the shortening of telomeres of circulating leukocytes in patients with obstructive sleep apnea [8]. Our previous study has shown that acute exposure to severe hypoxia at high altitude resulted in a significant elongation of telomeres in rat thymocytes and gastric mucosal cells [9, 10], but no significant change was observed in telomere length of peripheral white blood cells [11]. However, the effect of acute hypoxia at high altitude on the telomere length of heart and lung tissue cells and the underlying mechanism remain unclear.

In this study, we measured the telomere length of rat heart and lung tissue cells in response to acute exposure to severe hypoxia and the levels of telomerase reverse transcriptase (TERT), hypoxia-inducible factor (HIF-1α and HIF-2α), and reactive oxygen species (ROS), in order to elucidate their role in hypoxia-induced damage to heart and lung tissues.

## Methods

### Experimental animals and grouping

All animal experiments were approved by the Ethical Committee of Qinghai Provincial People’s Hospital. Forty male Wistar rats (6-week old) provided by the Experimental Animal Center of Lanzhou University (Lanzhou, China) were housed in a temperature-controlled environment at 25 °C with a 12-h light, 12-h dark cycle and had access to water and food ad libitum. The rats were randomized into control group (*n* = 10) and hypoxia group (*n* = 30). Rats in control group were kept at an altitude of 1500 m in Lanzhou city, while rats in hypoxia group were exposed to simulated hypoxia with an altitude of 5000 m in a low-pressure oxygen chamber (DYC-3000, 8 m × 3 m × 3 m; Guizhou Fenglei Aviation Machinery Co., Ltd., China) for 1 day (hypoxia-1 group; *n* = 10), 3 days (hypoxia-3 group; *n* = 10), and 7 days (hypoxia-7 group; *n* = 10), respectively. The left ventricular and right middle lobe tissues of each rat were collected for further analysis.

### Histological analysis

The left ventricular and right middle lobe tissues were collected and washed with normal saline, cut into pieces of 2 mm^3^, fixed with 10% paraformaldehyde solution, dehydrated in increasing grades of ethanol, embedded in paraffin, and subsequently sliced using a slicer. The tissue slices were stained with hematoxylin-eosin (HE), dehydrated, and sealed for observation under a microscope.

### The measurement of telomere length

The genomic DNA of rat heart and lung tissue cells was extracted using the DNA extraction kit (Beijing Tiangen Biotech Co., Led., China) according to the manufacturer’s instruction. The genomic DNA with an OD260/OD280 ratio of 1.8~ 2.0 and a concentration of ≥ 0.1 μg/μL was used. The DNA telomere length was determined by real-time polymerase chain reaction in a volume of 20 μL consisting of 10 μL of 2 × SYBR Premix Ex Taq, 2 μL of genomic DNA (25 ng/μL), and Tel rat-f and Tel rat-r primers, which were designed using Primer Premier 5.0 and synthesized by Invitrogen (Table 1). PCR reactions were performed in an ABI7500 RT-PCR system (Applied Biosystems, CA, USA) as follows: 95 °C 30 s, 95 °C 5 s, 60 °C 34 s, 40 cycles. The telomere length was expressed as the relative telomere/single copy gene (*T*/*S*) ratio, which could be calculated from the Ct values of telomeres and reference. ΔCt = Ct telomere − CtAT1; the relative *T*/*S* ratio = 2^−(ΔCt1 − ΔCt2)^ = 2 ^− ΔΔCt^, where ΔCtl and ΔCt2 are the ΔCt values of the experimental and control group, respectively.

**Table 1:** Primers used in this study

Primers	Sequence	Amplicon size (bp)
*Tel* rat-f	5′-GGT TTT TGA GGG TGA GGG TGA GGG TGA GGG TGA GGG T-3′.	
*Tel* rat-r	5′-TCC CGA CTA TCC CTA TCC CTA TCC CTA TCC CTA TCC CTA-3′.	
*AT1* rat-f	5′-ACG TGT TCT CAG CAT CGA CCG CTA CC-3′.	279
*AT1* rat-r	5′-AGA ATG ATA AGG AAA GGG AAC AAG AAG CCC-3′.	
*Tert* rat-f	5′-GAC ATG GAG AAC AAG CTG TTT GC-3′.	185
*Tert* rat-r	5′-ACA GGG AAG TTC ACC ACT GTC-3′.	
*Hif1a* rat-f	5′-CTA TGA CGT GCT TGG TGC TGA T-3′.	271
*Hif1a* rat-r	5′-CTG TAC TGT CCT GTG GTG ACT T-3′.	
*Epas1* rat-f	5′-TGACTTCACTCATCCTTGCGACCA-3′.	443
*Epas1*rat-r	5′-ATTCATAGGCAGAGCGGCCAAGTA-3′.	
*18S RNA* rat-f	5′-AGT GAT CCC CGA GAA GTT T-3′.	135
*18S RNA* rat-r	5′-GCT TTC CTC AAC ACC ACA T-3′.	

### The measurement of mRNA and protein levels of TERT, HIF-1α, and HIF-2α

The total RNA was extracted from rat heart and lung tissue cells using TRIzol RNA extraction kit (Invitrogen, USA). About 2 μg of total RNA with an A260/A280 of 1.9–2.0 was used as the template. The first strand cDNA was synthesized by reverse transcription. The primers of rat genes *Tert*, *Hif1a* (encoding HIF-1α), *Epas1* (encoding HIF-2α), and reference *Agtr1a* gene and 18S RNA were designed using Primer Premier 5.0 and synthesized by Invitrogen (Table 1). PCR reaction system (20 μL) consisted of 10 μL of 2 × SYBR Premix Ex Taq, 1 μL of first strand cDNA, and 1 μL of the upstream and downstream primers of *Tert*, *Hif1a*, *Epas1*, and reference 18S RNA. All PCR reactions were performed in an ABI7500 RT-PCR system under the following conditions for both target and reference genes: 50 °C 2 min, 95 °C 10 min, 95 °C 15 s, 60 °C 1 min, 40 cycles. The 2^−ΔΔCt^ method was used to calculate the mRNA levels of *Tert*, *Hif1a*, and *Epas1*. The total protein of rat heart and lung tissue cells was extracted using a tissue protein extraction kit (Beyotime Institute of Biotechnology, China). The protein content of TERT, HIF-1α, and HIF-2α was determined using ELISA kits (Wuhan USCN Business Co., Ltd., China) according to the manufacturer’s instruction.

### The measurement of ROS

The rat heart and lung tissues were accurately weighed, homogenized on ice, and centrifuged at 1000*g* for 10 min. Subsequently, the supernatant was collected for the measurement of ROS level using ROS detection kit (Nanjing Jiancheng Bioengineering Co., Ltd., China) according to the manufacturer’s instruction, and the results were expressed as the fluorescence intensity.

### Statistical analysis

All data were expressed as mean ± SD and analyzed using SPSS 17.0. Comparisons among groups were determined using one-way analysis of variance (ANOVA), followed by least significant difference (LSD) for multiple comparison. The bivariate correlation of TERT with telomere length, HIF-1α, and HIF-2α was performed. *p* value of < 0.05 was considered statistically significant.

## Results

### Changes in morphology of rat heart and lung tissues

**Fig. 1:**
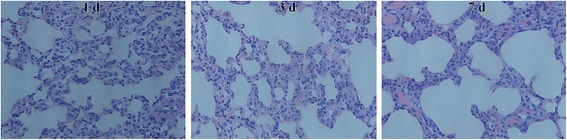
The morphology of rat lung tissues exposed to hypoxia for 1, 3, and 7 days. Control group: alveolar and pulmonary interstitial structure was normal without liquid exudation. Hypoxia-1 group: alveolar wall structure was normal, but there were some areas of telangiectasia congestion and mild pulmonary wall thickening of small arteries. Hypoxia-3 group: there were widening of alveolar wall, focal emphysema, focal alveolar hemorrhage, and slight thickening of the small arterial wall in the lungs. Hypoxia-7 group: the alveolar cavity was obviously deformed and smaller, the interstitial thickening and enlargement of the pulmonary interstitium, thickening of the small arterial wall, and the telangiectasia and congestion were obvious. HE staining (× 400)

**Fig. 2:**
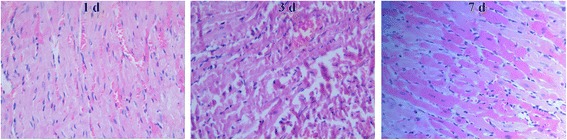
The morphology of rat heart tissues exposed to hypoxia for 1, 3, and 7 days. Control group: myocardial structure was normal, myocardial cells arranged in parallel, the nucleus was located in the center. Hypoxia-1 group: myocardial fiber arrangement was normal, but there were some inflammatory cell infiltration and muscle cell nucleus shrinkage and apoptosis. Hypoxia-3 group: there were mild myocardial fiber arrangement disorder, widened myocardial interstitium, cell edema, loose cytoplasm, rounded nucleus, and increased interstitial blood vessels. Hypoxia-7 group: there were severe myocardial fiber arrangement disorder, severe muscle fiber bundle damage, and large number of muscle cell necrosis; muscle cell boundary was unclear. HE staining (× 400)

Rats were exposed to simulated hypoxia with an altitude of 5000 m for 1, 3, and 7 days, respectively, and their heart and lung tissues were collected and stained with HE for the observation of morphology. It is apparent that as the duration of exposure to hypoxia increases, the heart and lung tissue injury gradually increased, characterized by the deformation and shrinking of alveoli; thickening of pulmonary interstitial tissues, telangiectasia, and obvious congestion (Fig. 1); broadening of myocardial interstitium, cellular edema, loose cytoplasm, and round nuclear contour; and increase in interstitial blood vessels (Fig. 2).

### Changes in telomere length in response to hypoxia

The telomere length of rat heart and lung tissue cells was determined by RT-PCR. The results showed that compared with control group, the telomere length was significantly longer in rats exposed to severe hypoxia for 1–7 days (*p <* 0.05). The telomere length increased with prolonged exposure, indicating that acute hypoxia can induce the elongation of telomeres of rat heart and lung tissue cells (Table 2).

**Table 2:** Telomere length of rat heart and lung tissue cells

Group	Sample	Telomere length of heart tissue	Telomere length of lung tissue
Control group	10	0.89 ± 0.16	1.10 ± 0.34
Hypoxia-1 group	10	1.57 ± 0.39^#^	2.07 ± 0.54^#^
Hypoxia-3 group	10	2.73 ± 0.73^#△^	3.96 ± 0.98^#△^
Hypoxia-7 group	10	4.11 ± 1.07^#△*^	5.02 ± 1.52^#△*^

#*p* < 0.05 vs. Control group; ^△^*p* < 0.05 vs. Hypoxia-1 group; *p < 0.05 vs. Hypoxia-3 group

### Changes in *Tert*, *Hif1a*, and *Epas1* mRNA levels in response to hypoxia

The mRNA levels of *Tert*, *Hif1a*, and *Epas1* of rat heart and lung tissue cells were determined by RT-PCR. The mRNA levels of *Tert* and *Hif1α* were significantly higher in rats exposed to hypoxia for 1–7 days than in control rats (*p <* 0.05) and also increased with prolonged exposure. However, the mRNA level of *Epas1* increased only in the hypoxia-7 group (Tables 3 and 4).

**Table 3:** *Tert*, *Hif1a*, and *Epas1* mRNA levels of rat heart tissues

Group	Sample	*Tert*	*Hif1a*	*Epas1*
Control group	10	0.96 ± 0.24	1.00 ± 0.18	0.96 ± 0.18
Hypoxia-1 group	10	2.25 ± 0.68^#^	1.77 ± 0.56^#^	1.00 ± 0.21
Hypoxia-3 group	10	3.63 ± 1.14^#△^	3.02 ± 0.92^#△^	0.96 ± 0.21
Hypoxia-7 group	10	4.98 ± 1.21^#△*^	4.69 ± 1.04^#△*^	1.49 ± 0.47^#^

#*p* < 0.05 vs. Control group; ^△^*p* < 0.05 vs. Hypoxia-1 group; *p < 0.05 vs. Hypoxia-3 group

**Table 4:** *Tert*, *Hif1a*, and *Epas1* mRNA levels of rat lung tissues

Group	Sample	*Tert*	*Hif1a*	*Epas1*
Control group	10	0.96 ± 0.22	0.94 ± 0.15	1.00 ± 0.19
Hypoxia-1 group	10	2.31 ± 0.63^#^	1.90 ± 0.38^#^	1.02 ± 0.12
Hypoxia-3 group	10	3.66 ± 1.17^#△^	2.90 ± 0.87^#△^	1.04 ± 0.26
Hypoxia-7 group	10	4.93 ± 1.07^#△*^	4.34 ± 1.17^#△*^	1.69 ± 0.77^#^

#*p* < 0.05 vs. Control group; ^△^*p* < 0.05 vs. Hypoxia-1 group; *p < 0.05 vs. Hypoxia-3 group

### Changes in TERT, HIF-1α, and HIF-2α protein levels in response to hypoxia

The protein levels of TERT, HIF-1α, and HIF-2α of rat heart and lung tissue cells were determined using ELISA. Tables 5 and 6 show that the changes in protein levels of TERT, HIF-1α, and HIF-2α in response to hypoxia coincided exactly with those of mRNA levels.

**Table 5:** TERT, HIF-1α, and HIF-2α protein levels of rat heart tissues

Group	Sample	TERT (ng/ml)	HIF-1α (ng/ml)	HIF-2α (ng/ml)
Control group	10	7.23 ± 1.81	16.02 ± 3.14	17.09 ± 4.53
Hypoxia-1 group	10	12.68 ± 3.43^#^	22.81 ± 4.77^#^	18.22 ± 3.81
Hypoxia-3 group	10	17.91 ± 2.69^#△^	27.89 ± 5.82^#△^	16.55 ± 6.08
Hypoxia-7 group	10	21.56 ± 3.22^#△*^	33.08 ± 4.74^#△*^	23.57 ± 6.09^#^

#*p* < 0.05 vs. Control group; ^△^*p* < 0.05 vs. Hypoxia-1 group; *p < 0.05 vs. Hypoxia-3 group

**Table 6:** TERT, HIF-1α, and HIF-2α protein levels of rat lung tissues

Group	Sample	TERT (ng/ml)	HIF-1α (ng/ml)	HIF-2α (ng/ml)
Control group	10	7.77 ± 2.21	9.48 ± 2.25	18.72 ± 4.58
Hypoxia-1 group	10	12.97 ± 3.53^#^	13.36 ± 3.00^#^	20.87 ± 3.97
Hypoxia-3 group	10	20.33 ± 4.28^#△^	18.33 ± 3.76^#△^	19.19 ± 3.01
Hypoxia-7 group	10	25.11 ± 6.01^#△*^	23.50 ± 5.40^#△*^	26.63 ± 7.75^#^

#*p* < 0.05 vs. Control group; ^△^*p* < 0.05 vs. Hypoxia-1 group; *p < 0.05 vs. Hypoxia-3 group

### Changes in ROS production in response to hypoxia

The ROS levels of heat and lung tissue cells were determined by chemical fluorometric methods. The results showed that compared with control group, ROS level increased significantly in each hypoxia group (*p <* 0.05) and increased further with the increase of exposure duration (Table 7).

**Table 7:** ROS levels of rat heart and lung tissues

Group	Sample	ROS level of heart tissue	ROS level in lung tissue
Control group	10	2688.11 ± 137.29	1965.26 ± 85.37
Hypoxia-1 group	10	6974.25 ± 269.34^#^	4792.65 ± 176.33^#^
Hypoxia-3 group	10	15,631.47 ± 413.98^#△^	10,669.46 ± 328.64^#△^
Hypoxia-7 group	10	27,356.19 ± 721.36^#△*^	21,154.63 ± 593.71^#△*^

#*p* < 0.05 vs. Control group; ^△^*p* < 0.05 vs. Hypoxia-1 group; *p < 0.05 vs. Hypoxia-3 group

### The correlation of TERT with HIF-1α and HIF-2α levels and telomere length

Table 8 shows that TERT protein levels of heart and lung tissue cells were positively correlated with HIF-1α protein level (*r* = 0.678–0.736, *p* < 0.001) but not with HIF-2α protein level (*p* > 0.05). Table 9 shows that TERT mRNA levels of heart and lung tissue cells were positively correlated with *Hif1a* mRNA level and telomere length (*r* = 0.843–0.913, *p* < 0.001) but not with *Epas1* mRNA level (*p* > 0.05).

**Table 8:** Correlation of TERT protein level with HIF-1α and HIF-2α protein levels in heart and lung tissues

		HIF-1α protein level	HIF-2α protein level
TERT of heart tissue	*r*	0.678	0.295
	*p*	< 0.001	0.064
TERT of lung tissue	*r*	0.736	0.256
	*p*	< 0.001	0.111

**Table 9:** Correlation of TERT mRNA level with *Hif1a* and *Epas1* mRNA levels and telomere length in heart and lung tissues

		*Hif1a* mRNA level	*Epas1* mRNA level	Telomere length
TERT of heart tissue	*r*	0.913	0.283	0.843
	*p*	< 0.001	0.077	< 0.001
TERT of lung tissue	*r*	0.868	0.274	0.879
	*p*	< 0.001	0.087	< 0.001

## Discussion

In this study, we found that increasing exposure duration to hypoxia can damage rat heart and lung tissue cells and increase ROS production and telomere length. Cataldi et al. showed that hypoxia by producing a large quantity of ROS could damage cellular components through the oxidation of DNA, proteins, and lipids, resulting in telomere shortening of myocardial cells and cellular senescence and apoptosis [12]. Similarly, Roy et al. found that cardiac fibroblasts isolated from adult murine ventricle cultured in hyperoxia exhibited higher levels of ROS production and lowered telomerase activity [13]. In contrast, Xu et al. showed that hypoxia could induce myocardial cells to express TERT, resulting in increased telomere length and proliferation of myocardial cells [14]. Previous studies have also shown that hypoxia triggers a cellular adaptive response that is primarily mediated by HIF-1 and extends the life span of vascular smooth muscle cells through telomerase activation [7, 15]. The discrepancy of these results may be explained by the differences in cell lines and hypoxia exposure conditions and duration. Our results are in agreement with previous studies on hypoxia-induced telomere elongation [7, 14, 15].

In addition, mRNA and protein levels of TERT and HIF-1α were significantly higher in rats exposed to hypoxia and increased with the exposure duration. However, the situation was different for HIF-2α, which increased only in rats exposed to hypoxia for 7 days. In addition, TERT level was positively correlated with telomere length and HIF-1α mRNA and protein levels but not with HIF-2α mRNA and protein levels. These data suggest that HIF-1α may play a more important role in the initial response to hypoxia and HIF-2α may function in later response to hypoxia [16].

Telomerase is a ribonucleoprotein complex composed of two essential components, TERT protein and telomerase RNA (TERC) [17]. The primary function of telomerase is to elongate telomeres by adding a species-dependent telomere repeat sequence to the 3′ end of telomeres. TERT plays an important role in regulating telomerase activity [18–20]. HIF-lα is the most important regulatory factor in the response of cells to hypoxia [21]. HIF-1α is the major activating transcription factor to induce TERT transcription, and hypoxia can induce HIF-1α in various cells, which in turn can regulate the transcription of TERT and increase TERT expression and telomerase activity [22, 23]. The exposure to hypoxia resulted in ROS production, which can increase the transcription of HIF-1α by inducing PI3K/AKT and ERK phosphorylation [24]. Our findings of positive relationship between telomere length and TERT and HIF-1α levels indicate that acute exposure to severe hypoxia may directly induce the expression of HIF-1α or indirectly through the production of a large quantity of ROS to upregulate TERT expression.

## Conclusions

Acute exposure to severe hypoxia causes damage to rat heart and lung tissues due to the production of a large quantity of ROS but also increases telomere length and adaptive response by upregulating TERT expression through HIF-1α, which can protect heart and lung tissue cells from fatal damage. Human physiological adaptation to high altitude may also involve the upregulation of TERT expression and telomere elongation. TERT may be a potential target for the prevention and treatment of high-altitude sickness in human.

## Abbreviations

HIF-1α: Hypoxia-inducible factor1α
HIF-2α: Hypoxia-inducible factor 2α
ROS: Reactive oxygen species
TERT: Telomerase reverse transcriptase
